# Multifrequency STD NMR Unveils the Interactions of Antibiotics With *Burkholderia multivorans* Biofilm Exopolysaccharide

**DOI:** 10.3389/fmolb.2021.727980

**Published:** 2021-09-16

**Authors:** Ridvan Nepravishta, Serena Monaco, Marco Distefano, Roberto Rizzo, Paola Cescutti, Jesus Angulo

**Affiliations:** ^1^ School of Pharmacy, University of East Anglia, Norwich, United Kingdom; ^2^ Department Life Sciences, University of Trieste, Trieste, Italy; ^3^ Department of Organic Chemistry, Faculty of Chemistry, University of Seville, Seville, Spain; ^4^ Instituto de Investigaciones Químicas (CSIC-US), Seville, Spain

**Keywords:** STD NMR, multifrequency STD NMR, exopolysaccharides, biofilms, Burkholderia multivorans

## Abstract

Biofilms confine bacterial cells within self-produced matrices, offering advantages such as protection from antibiotics and entrapment of nutrients. Polysaccharides are major components in these macromolecular assemblies, and their interactions with other chemicals are of high relevance for the benefits provided by the biofilm 3D molecular matrix. NMR is a powerful technique for the study and characterization of the interactions between molecules of biological relevance. In this study, we have applied multifrequency saturation transfer difference (STD) NMR and DOSY NMR approaches to elucidate the interactions between the exopolysaccharide produced by *Burkholderia multivorans* C1576 (EpolC1576) and the antibiotics kanamycin and ceftadizime. The NMR strategies presented here allowed for an extensive characterization at an atomic level of the mechanisms behind the implication of the EpolC1576 in the recalcitrance phenomena, which is the ability of bacteria in biofilms to survive in the presence of antibiotics. Our results suggest an active role for EpolC1576 in the recalcitrance mechanisms toward kanamycin and ceftadizime, though through two different mechanisms.

## Introduction

In all living organisms, several strategies have been developed with regard to intercellular communication and mechanical or xenobiotic defense. At the molecular level, carbohydrate–carbohydrate or carbohydrate–protein interactions are essential parts of these biological processes. Specifically, in bacterial infections, these interactions are of paramount importance in the early stages of cell–host adhesion or in the bacterial collectivity life modality called biofilm (BF) (Flemming et al., 2016). With regard to the latter, a relevant way of bacterial colony life is their growth in self-produced matrices having a gel-like structure typical of the BF lifestyle. The composition of the matrices is chemically heterogeneous, including proteins, exopolysaccharides (Epols), and DNA, with Epols as the most abundant part of the BF and important for the matrix’s mechanical stability. The chemical structure of BF’s Epols is known only for a few bacterial species; however, depending on the type of Epol produced by the bacteria, it is presumed that either polar or hydrophobic non-covalent interactions might be, in general, involved in the resulting 3D assembly (Flemming, 2016). The internal architecture of Epols and their detailed mechanisms of interactions conferring mechanical stability to the biofilm still remain, up to date, elusive.

BF matrices take part actively in interactions with molecules of the surrounding environment (Limoli et al., 2015), and in particular, Epols have been shown to bind small molecules (Worthington et al., 2012), suggesting a possible active participation of Epols in many processes positively influencing bacterial life. Globally, the molecular interactions between Epols and small molecules constitute a network of molecular recognition processes that have great importance for the colony, mainly regarding nutrition, defense, and cell–cell communication. In fact, considering the nutrition aspect, the interactions of nutrients with the BF lead to a slow and constant diffusion of these molecules toward the colony. With regard to defense, recalcitrance, a multifactorial reversible defense phenomenon, confers non-susceptibility to bacteria toward antibiotics that disappear when the BF is disrupted. As with the nutrients, the interactions of antibiotics with the BF significantly impact their effectiveness, and a corresponding reduction in penetration to the cells has been demonstrated to be one of the mechanisms contributing to recalcitrance (Hall and Mah, 2017).

**SCHEME 1:**
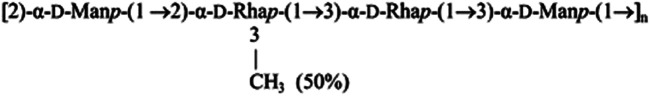
Repeating unit of the exopolysaccharide produced by *B. multivorans* C1576 (EpolC1576).

In this study, we wanted to unveil the molecular basis for some of the interactions that underlie the involvement of Epols in the defense mechanisms of bacteria, such as recalcitrance, demonstrating that the polysaccharide extracted from BF produced by *Burkholderia multivorans* reference strain C1576 (hereafter EpolC1576) interacts with ceftazidime (a cephalosporin antibiotic) and kanamycin (an aminoglycoside antibiotic) by two completely different molecular mechanisms. *B. multivorans* is part of the so-called *Burkholderia cepacia* complex, a family of bacteria; many of them involved in serious lung infections in cystic fibrosis patients that might evolve in premature death following severe decline in the lung function (Scoffone et al., 2017). *B. multivorans* on Mueller–Hinton agar produces a rhamno-mannan Epol containing α-D-rhamnose and α-D-mannose residues in equimolar ratios, with 50% *O*-methyl substitution on C-3 of the 2-linked rhamnose residues (Scheme 1) (Dolfi et al., 2015).

Accessing the possible mechanisms that can contribute to recalcitrance at the molecular level is not trivial. Very few biophysical techniques can achieve a depth in structural information at an atomic level without losing the focus on the dynamical interactions between the small molecule and the exopolysaccharide. Here, we hypothesized that the ^1^H saturation transfer difference (STD) NMR spectroscopy (Mayer and Meyer, 1999) could provide molecular level details of the interactions of BF-forming exopolysaccharides and small molecules. ^1^H STD NMR is the technique of choice for studies typically focusing on the interactions between a small ligand and a protein. Relying on a fast exchange kinetics regime between the free and bound states of the ligand, it gives powerful insights about the binding epitope (Mayer and James, 2004) and dissociation constant (K_D_) (Angulo et al., 2010), and, as lately shown, also about ligand orientation in the binding site (Monaco et al., 2017). Inspirational to our experimental setup in this study was the application of the selective frequency saturation transfer difference technique applied to the binding of small molecules to DNA (Di Micco et al., 2005; Martin et al., 2005; Souard et al., 2008). Differently to other NMR techniques, the strength of STD NMR is based on the fact that there is virtually no limitation on the upper limit of the molecular weight of the receptor. Based on this strength, many groups have explored the use of STD NMR to characterize the interaction of small molecules with viruses (Benie et al., 2003), receptors embedded at the outer region of the eukaryotic cell (Airoldi et al., 2011), and, lately, also interaction of small molecules with soft mater, such as hydrogels (Calabrese et al., 2019).

## Materials and Methods

### Bacterial Strain, Media, and Chemicals

*B. multivorans* C1576 (LMG 16660) is a reference strain that belongs to the panel of *B. cepacia* complex strains (EP1), and it was purchased from the BCCMTM bacteria collection. Mueller–Hinton Broth (MHB) and Bacto Agar were purchased from Difco Laboratories. The fluorescent probes 1-anilino-8-naphthalene-sulfonate sodium salt (Na + ANS-) and 2-(p-toluidinyl)-6-naphthalene sulfonate potassium salt (K + TNS-) as well as antibiotics kanamycin sulfate salt (Kanamycin A) and ceftazidime were purchased from Sigma-Aldrich.

Biofilms of *B. multivorans* C1576 were developed on cellulose membranes (Sigma, cutoff 12.400 Da) treated as follows: membranes were cut in circles to fit Petri dishes, boiled in 5% Na2CO3 solution for 15 min and then boiled in water for 15 min, subjected to autoclave sterilization, and laid on Petri dishes containing the MH agar medium. Membranes were extended all over the plate, and surplus water was let to evaporate under the hood. An overnight liquid culture of *B. multivorans* C1576 was diluted to obtain a cell suspension having 0.13 OD at 600 nm, and three aliquots of 10 µl of the cell dilution were transferred on the membranes. The same liquid medium was used for the overnight culture of *B. multivorans* C1576 and for Petri dish filling. After 7 days of incubation at 30°C, the biofilm on the membranes was recovered with sterile 0.9% NaCl, and then the cell suspension was added with NaOH to reach a final concentration of 0.1 M and incubated at room temperature, with shaking for 3 h to dismantle the biofilm. The cell suspension was then centrifuged at 40,000 g at 4°C for 30 min, and the supernatant, containing soluble EpolC1576, was recovered and dialyzed against distilled water using a 12–14 kDa molecular weight cutoff (MWCO, SERVA Electrophoresis GmbH, Heidelberg, Germany) membrane for 72 h at 25°C. The solution recovered from a dialysis tube was cooled in ice and added with trichloroacetic acid (20% w/v final concentration) to precipitate proteins and nucleic acids. After 30 min of incubation, the solution was centrifuged 40,000 g for 30 min at 4 °C, and the supernatant was collected and dialyzed against distilled water using a 12–14 kDa MWCO membrane for 72 h at 25°C. The dialysate was cooled in ice and added with 4 volumes of 96% cold ethanol, and the mixture was placed for 24 h at −20°C to separate the polysaccharide from lipids. The solution was then centrifuged at 40,000 g for 30 min at 4°C, and the pellet consisting of polysaccharide was resuspended in Milli-Q water and dialyzed against the same for 72 h at 4°C using a 12–14 kDa MWCO membrane. The remaining retentate was taken to neutral pH, filtered through a 0.22-µm pore size filter (KX Sterile Syringe Filter, Kinesis), and lyophilized. The purity of the EpolC1576 sample used in STD NMR experiments was assessed by means of ^1^H NMR, which also confirmed the primary structure (Dolfi et al., 2015, 411, 42-48.). The full ^1^H NMR spectrum is showed in Supplementary Figure S1; the integration values of anomeric proton peaks and rhamnose methyl group are in perfect agreement with the structure. No signals other than those pertaining to EpolC1576 are present in the spectrum, thus indicating that the sample was suitable to STD experiments. EpolC1576 was exchanged three times with 99.9% D_2_O (Sigma-Aldrich) by lyophilization and finally dissolved in 0.6 ml of 99.9% D_2_O before recording a ^1^H NMR spectrum on a 500-MHz Varian spectrometer operating at 50°C.

### STD NMR Experiments

All the NMR experiments were conducted using a Bruker Avance II 500 MHz machine in 20 mM Tris–d11 D_2_O buffer pH 7.5 at 298 K. 1D ^1^H STD NMR spectra (Mayer and Meyer, 1999) were acquired with SW of 16 ppm using a TD of 32 K data points using a recycling delay (D1) of 6 s with 64 scans for epitope mapping calculation. The Bruker library pulse stdiffesgp.3 was used for all the STD NMR experiments applying a train of 50-ms Gaussian-shaped pulse at 40 ppm (off-resonance spectra) and 3.2, −1.0 ppm for ANS while 1.22 and −1.0 ppm for kanamycin (on-resonance spectra). The residual water signal was suppressed by the excitation sculpting technique, while the Epol signals where possible were canceled using a 40-ms T1ρ filter. In order to obtain the epitope mappings for average orientation of the small molecules, the saturation time was varied from 0.5 to 6 s for each chosen saturation frequency. The results obtained were fitted using the equation STD_(tsat)_ = STD_max_ (1-exp (k_sat_*t_sat_)), where STD_(tsat)_ = I_0_-I/I_0_. Here, “I_0_” is the intensity of the chosen peak measured from “off-resonance” spectra, while “I” is the intensity of the same peak from the so-called “on-resonance” spectra. STD_0_ can be obtained easily using the equation STD_0_ = k_sat_ * STD_max_. The data were then normalized to the highest STD_0_ in order to obtain STD_0_ (%). The STD_0_ (%) factor was classified as strong for values between 81–100%, as intermediate for values between 50–80%, and weak for values obtained between 0–49%. This scale assures the analysis of proximity, on average, of the ligand atom to the interface with the EpolC1576 and is derived for each saturation frequency.

The DOSY experiment with the aim to characterize the products of degradation was performed using the Bruker library pulse sequence ledbpgppr2s (Wu et al., 1995). The SW for the experiment was set to 16 ppm, the big delta (Δ) was set to 100 ms, the little delta (δ) was set to 3 ms, while D1 was set to 2 s. A linear gradient spanning from 5 to 95% in 16 points was used. The data were fitted using the T1/T2 module and transformed in 2D DOSY spectra using the DOSY routine for topspin 3.6.

### Sample Preparation

The interaction experiments between ANS and EpolC1576 were conducted at a concentration of 5 mM and 0.5 mg/ml. Not knowing the exact MW of EpolC1576, the ratio was chosen empirically as the best ratio to obtain a good STD NMR signal while attempting to maintain the EpolC1576 concentration as low as possible. For the interaction and competition studies between ANS and kanamycin or ceftazidime, the same concentrations as for interaction experiments were used for EpolC1576 and ANS (0.5 mg/ml and 5 mM), while the concentration of kanamycin or ceftazidime was changed from 0.5 to 2 mM. The change in the intensity of the STD NMR for ANS peaks was monitored accordingly (see the text). Degradation studies with the aim to focus on the small amounts of the degradation product (pyridine) required higher amount of ceftazidime that in this case was used at a concentration of 6 mM, while the EpolC1576 was kept at the usual concentration of 0.5 mg/ml. All the described experiments were performed in 20 mM Tris–d11 D_2_O buffer pH 7.5 at 298 K. All control STD NMR experiments in order to access direct saturation for ANS, ceftazidime, and kanamycin were performed at the concentration of 5, 6, and 2 mM, respectively, in the absence of EpolC1576 in 20 mM Tris–d11 D_2_O buffer pH 7.5 at 298 K. No direct saturation was observed.

## Results and Discussion

### STD NMR Is a Sensitive Technique to Detect and Characterize the Interactions of the Exopolysaccharide With Small Molecules

We first wanted to probe the feasibility of the STD NMR approach for this type of interaction. The hydrophobic properties of EpolC1576 have been previously studied through its interactions with small molecules as 2-(p-toluidinyl)-naphthalene-6-sulfonate (TNS) and 1-anilinonaphthalene-8-sulfonate (ANS) (Kuttel et al., 2017). In that study, interactions of both compounds with EpolC1576 were demonstrated by simple observation of line broadening in the NMR spectra of ANS and TNS in the presence of EpolC1576, in contrast to the sharp lines observed in the negative control experiments in the presence of dextran, suggesting interactions taking place with EpolC1576 and them being within the so-called fast exchange regime. These results encouraged us to run a preliminary exploration of the capability of the ^1^H STD NMR technique for the first time, to the best of our knowledge, for the detection of interactions of a bacterial Epol with small molecules.

**FIGURE 1:**
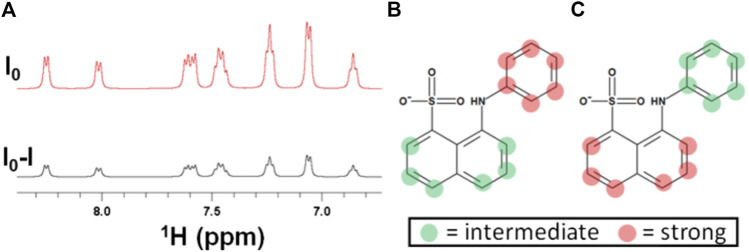
Detection and differential-binding epitope characterization of the interaction of ANS with EpolC1576. **(A)** STD NMR spectra of ANS (5 mM) in the presence of EpolC1576. **(B)** ANS-binding epitope obtained by selectively saturating EpolC1576 at +1.22 ppm and **(C)** by selectively saturating EpolC1576 at +3.22 ppm. A comparison of **(B)** and **(C)** indicates that the phenyl ring of ANS orients, on average, toward the CH_3_ moiety of EpolC1576. Intermediate STD_0_ (%) = 50–80% and strong STD_0_ (%) = 80–100%.

Figure 1 demonstrates that STD NMR detection of the interaction of ANS with EpolC1576 in solution is possible as strong STD signals were obtained in the difference spectrum (Figure 1A), in contrast to the absence of STD signals for a control sample containing ANS and dextran (Supplementary Figure S2). In fact, the excellent sensitivity of the resulting spectra allowed us to undertake further structural investigations to elucidate if any preferential orientation of the ligand ANS is taking place within the areas of interaction with EpolC1576. To that aim, we employed a slightly modified application of the multifrequency STD NMR methodology termed the DEEP-STD NMR approach (Monaco et al., 2017). It is important to note that we are not dealing here with a classical one-site binding type of interaction, but, instead, multiple areas of interaction are expected due to the repetitive pattern of the polysaccharide primary structure (Scheme 1). For this reason, we proposed to resort to the initial growth rate STD_0_ (%) values and introduce the concept of “average orientation” in the time scale of STD NMR spectroscopy. Briefly, we carried out a comparative analysis of the binding epitope mappings of ANS obtained from a pair of STD NMR experiments using two different selective saturation conditions of EpolC1576: one at frequency +3.22 ppm where we directly irradiate the CH protons of the polysaccharide and another at +1.22 ppm corresponding to CH_3_ moiety of EpolC1576 (Figures 1B,C).

Under these conditions, significant differences in the epitope mappings were observed and the results are interpreted as follows: those protons showing the highest STD_0_ (%) values (in red dots in Figures 1B,C) are oriented, on average, toward the interface of binding encoded by the particular frequency of saturation used. Using this approach, we were able to conclude that ANS interacts with a “hydrophobic” region of the EpolC1576 orienting its phenyl ring toward the CH_3_ group of EpolC1576 while orienting its naphthyl ring, on average, toward the CH moieties presumably of the rhamnose residues. These results are in excellent agreement with previous molecular simulations showing that the hydrophobic domains along EpolC1576 are associated primarily with contiguous rhamnose residues with partial *O*-methyl substitutions (Kuttel et al., 2017).

### STD NMR Study of the Interactions With Antibiotics: Ceftazidime and Kanamycin

**FIGURE 2:**
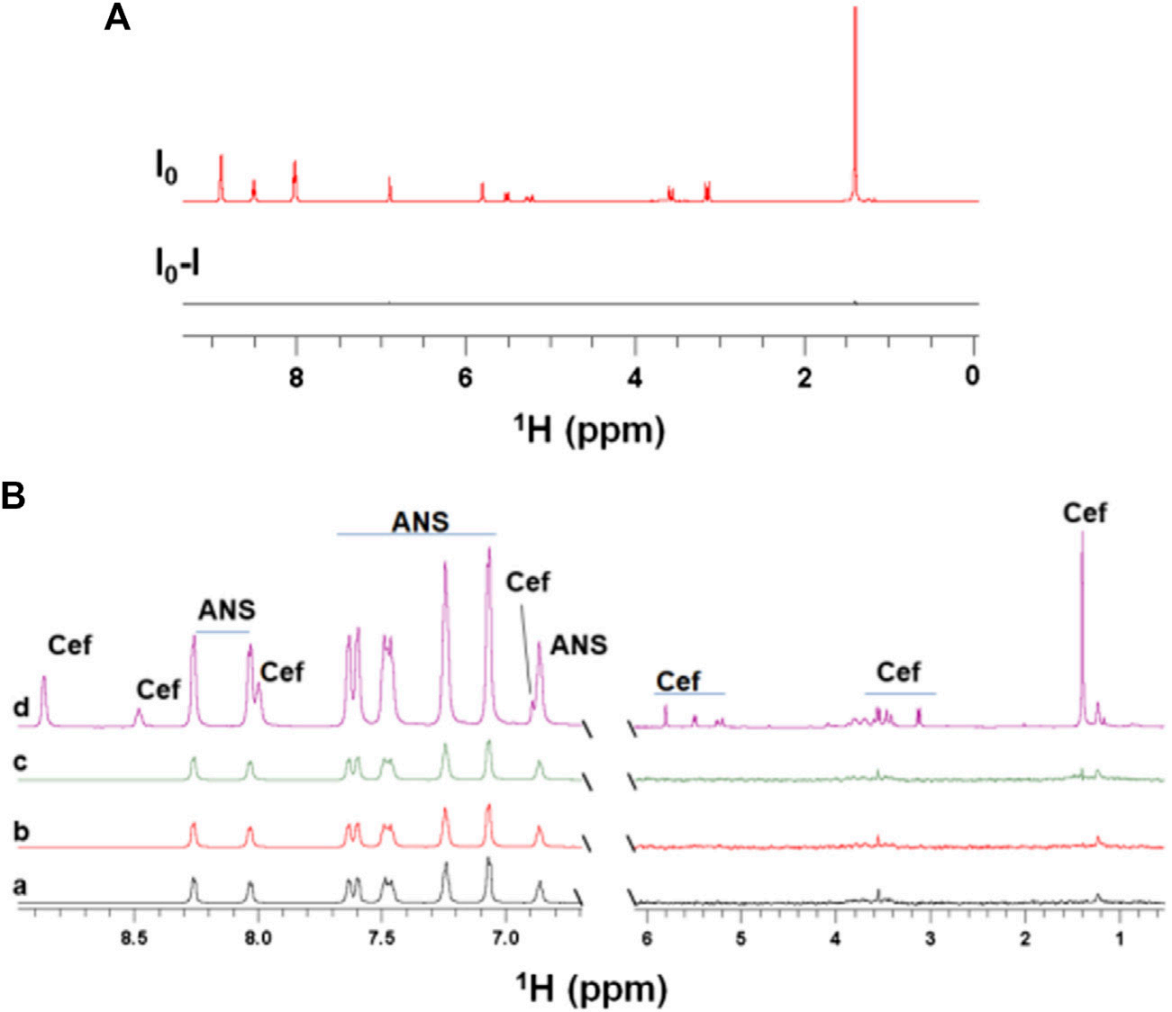
STD NMR does not detect interactions between EpolC1576 and ceftazidime. **(A)** ^1^H STD NMR spectra of ceftazidime in the presence of EpolC1576, suggesting that no interaction occurs. **(B)** Competition studies of ceftazidime with ANS. STD NMR spectra of ANS (5 mM) in the absence (a) and in the presence of increasing concentrations of ceftazidime in (b) (0.5 mM), (c) (2 mM), and (d) reference spectra.

After the successful application of STD NMR to characterize the interactions of EpolC1576 with ANS, we decided to investigate the hypothesis that different domains on the Epol could facilitate complexation with antibiotics and slow down their diffusion through the biofilm toward the bacterial cell surface. We used ^1^H STD NMR to characterize the interaction of the two structurally different antibiotics ceftazidime and kanamycin with EpolC1576. To this aim, we opted for two main strategies: the direct assessment of the interaction using the slightly modified DEEP-STD NMR method and an STD NMR indirect assessment by using ANS as a probe in competition assays (Wang et al., 2004). We first studied the interaction of ceftazidime with EpolC1576. As shown in Figure 2A, no signals were observed in the STD NMR experiments, which, in a first glance, would support that ceftazidime does not interact with EpolC1576.

To ensure that this result was not depending on the NMR methodology used (in fact, very slow exchange mechanisms would lead to similar STD NMR negative results), we ran a ^1^H STD NMR competitive approach between ceftazidime and ANS. As it is shown in Figure 2B, the absence of the peaks of the ceftazidime in the STD NMR spectra and, what is more, the absence of any impact on the STD factors of ANS indicate that ceftazidime is not a strong binder. Taken together, these data suggest that no binding occurs between ceftazidime and EpolC1576 in the time scale of the observation of the NMR techniques used.

However, the absence of binding of ceftazidime to EpolC1576 does not preclude an interaction that can occur at time scales not detectable by the used NMR spectroscopic techniques. For that, we decided to follow a functional approach. In fact, it is known that cephalosporines undergo degradation when in neutral or basic media so that affecting the kinetics of that degradation process can constitute a mechanism of disturbing the antibiotic efficacy (Vilanova et al., 1993). Thus, we decided to investigate whether the presence of EpolC1576 can impact the degradation rate of the antibiotic, supporting a way of EpolC1576-induced ceftazidime recalcitrance. In order to confirm this hypothesis, two samples of ceftazidime in the presence and the absence of EpolC1576 in 20 mM Tris–d11 at pH 7.5 were incubated at 298 K.

**FIGURE 3:**
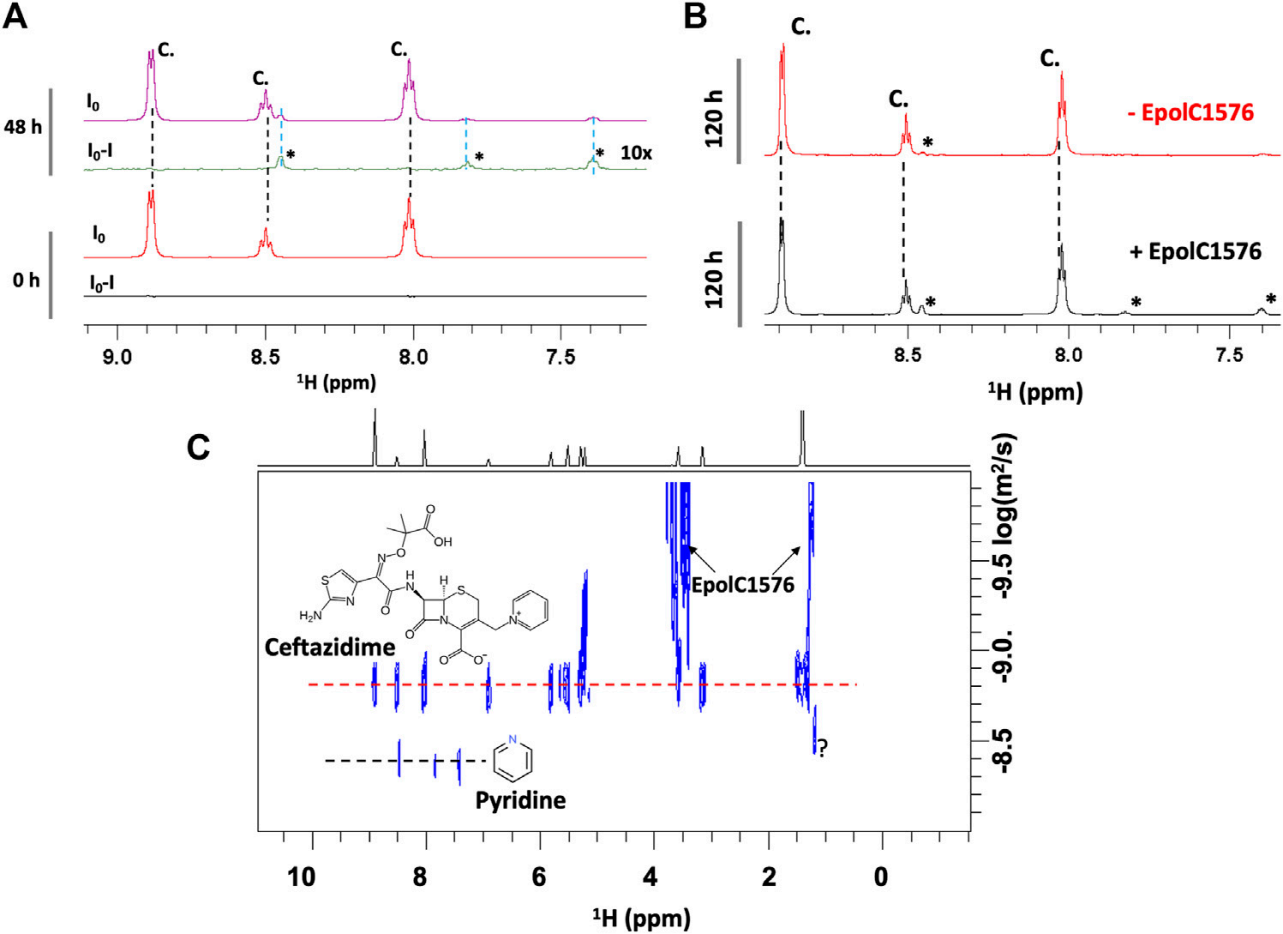
EpolC1576 accelerates the degradation of ceftazidime. **(A)** ^1^H STD NMR spectra at 0 and 48 h of ceftazidime (6 mM) in the presence EpolC1576. Only the degradation product interacts with EpolC1576. **(B)** ^1^H NMR spectra of ceftazidime in the presence and absence of EpolC1576 after 120 h in 20 mM Tris pH 7.5 at 298 K. The degradation product peaks are clearly visible in the presence of EpolC1576 but only slightly noticeable in its absence. **(C)** DOSY NMR spectra revealing the low molecular weight degradation product compared to ceftazidime. The degradation product was assigned as pyridine. The peak labeled with “?” is another degradation product yet not characterized.

In fact, a significant increase in the concentration of degradation products was observed in the presence of EpolC1576 after 48 and 120 h of incubation (Figure 3). This suggests that the EpolC1576 might contribute to the recalcitrance toward ceftazidime through degradation of the antibiotic (Figure 3). Interestingly, STD NMR experiments showed that, in contrast to ceftazidime, the degradation product interacts directly with the EpolC1576, as shown in Figure 3B. Further analysis using DOSY NMR (Figure 3C) revealed that the new species presents a set of three resonances in the aromatic region and a faster diffusion constant compared to ceftazidime. This is indicative of a molecule with smaller molecular weight. The set of NMR resonances of this low molecular weight species was confronted with the ^1^H NMR spectra of known degradation products of ceftazidime and was assigned to pyridine (Vilanova et al., 1993).

**FIGURE 4:**
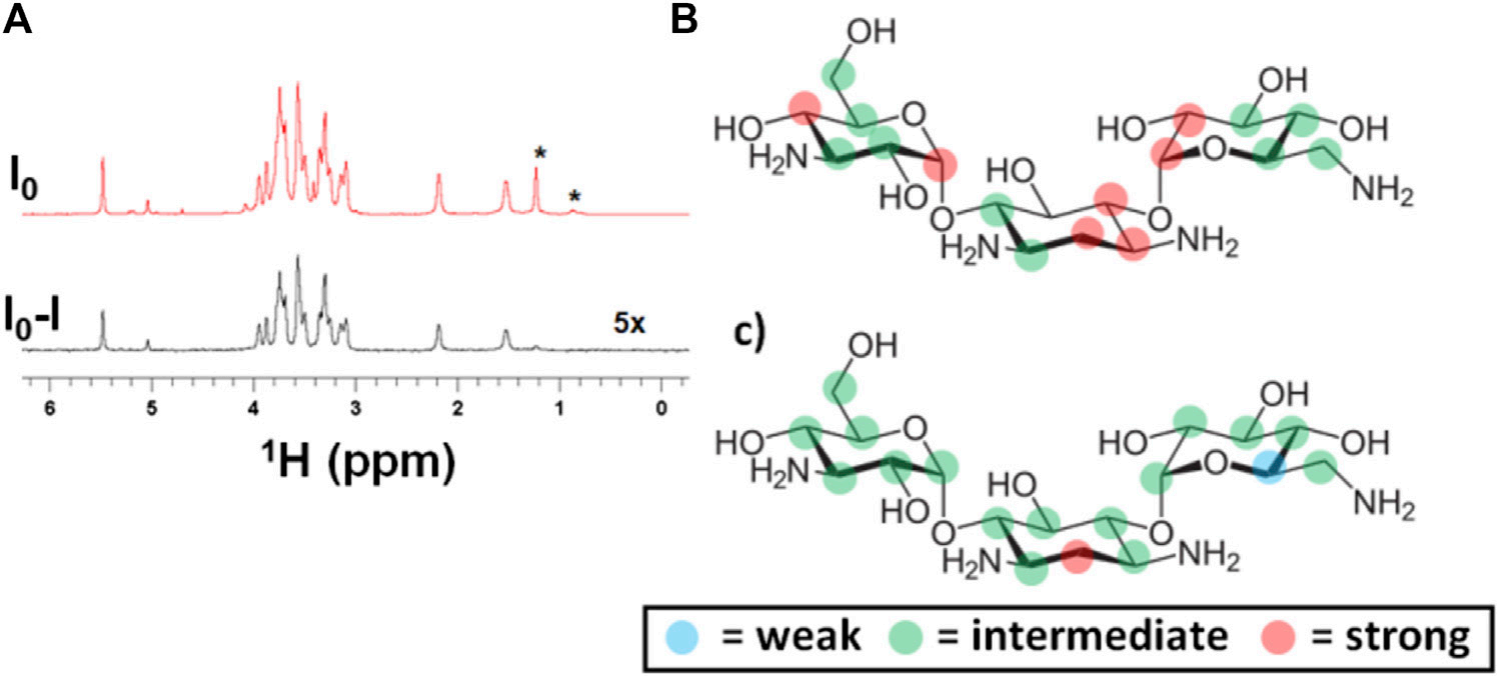
Detection and differential binding epitope characterization of the interaction of kanamycin with EpolC1576. **(A)** (top) reference and (bottom) STD NMR spectra demonstrating the highly sensitive detection of the interaction of kanamycin (2 mM) with EpolC1576 by selectively saturating EpolC1576 at −1.0 ppm, **(B)** binding epitope mapping of kanamycin by selectively saturating EpolC1576 at −1.0 ppm, and **(C)** binding epitope mapping by selectively saturating EpolC1576 at +1.2 ppm. The resonances labeled with the asterisk (*) belong to the EpolC1576. Weak STD_0_ (%) = 0–49%, intermediate STD_0_ (%) = 50–80%, and strong STD_0_ (%) = 81–100%.

The study was then focused on the interaction of the other antibiotic, kanamycin, with EpolC1576, following a similar strategy. In this case, the ^1^H STD NMR experiment clearly demonstrated that kanamycin interacts with EpolC1576 in a solution (Figure 4A). Additionally, the data suggested that EpolC1576 forms a macromolecular network in water, with extremely broadened NMR signals and very efficient saturation even at −1 ppm.

To further characterize the interaction of kanamycin with EpolC1576, a complete analysis of the orientation of the antibiotic in the area of interaction using the slightly modified DEEP-STD NMR approach was performed, as previously described (Di Micco et al., 2005; Martin et al., 2005; Souard et al., 2008; Monaco et al., 2017). Again, the interpretation of the epitope mappings obtained (Figures 4B,C) was based on the concept of “average orientation” of the protons due to the absence of a classic one-binding site interaction. As in the case of ANS, the protons showing the highest STD_0_ (%) values (red dots in Figures 4B,C) are oriented, on average, toward the interface of interaction with the EpolC1576. With this technique, we were also able to characterize which EpolC1576 monomers are found at the interface of interaction and are responsible for the transfer of saturation. Indeed, they can be derived from the saturation frequency used: CH and CH_3_ moieties at −1 ppm (nonselective saturation frequency) and CH_3_ moiety at 1.22 ppm (selective saturation frequency). The differences between the two epitope maps obtained (Figures 4B,C) suggest that the central ring of the molecule of kanamycin is oriented, on average, toward the CH_3_ moiety of rhamnose monomers, in a likely similar location as the benzyl ring of ANS.

We then further characterized the interaction areas of EpolC1576 with kanamycin and investigated if those areas indeed overlap with the interaction areas of ANS by performing a ^1^H STD NMR competitive study. For this, an STD NMR spectrum at 4 s saturation time of a 0.5 mg/ml EpolC1576 sample containing 5 mM ANS was acquired. Shortly after, kanamycin at different concentrations was added to this solution. The ^1^H STD NMR spectra obtained were monitored for the presence of kanamycin peaks and for the impact on STD NMR factors of ANS.

**FIGURE 5:**
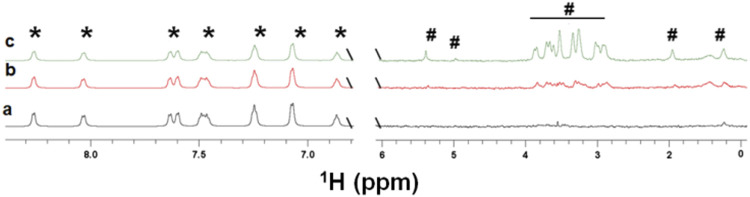
Competition study of kanamycin with ANS. STD NMR spectra of ANS (5 mM) in the absence a) and in the presence of increasing concentrations in b (0.5 mM) and c (2 mM) of kanamycin. The presence of the peaks of kanamycin shows that the antibiotic is interacting with EpolC1576. However, reductions, on average, of 4 and 20% of STD factors for ANS were observed for samples in b and c (see the text) confirming its displacement. The resonances labeled with the (*) symbol belong to the ANS molecule, while those labeled with the (^#^) symbol belong to the kanamycin molecule.

The spectra (Figure 5 b,c) show that kanamycin binds to the EpolC1576 in the presence of ANS. Furthermore, an increase in the concentration of kanamycin (Figure 5 b,c) led to a progressive reduction in STD factors of ANS (4 and 20% reduction for 0.5 and 2 mM of kanamycin, respectively). Taken together, these data strongly support that kanamycin displaces ANS from its interaction sites and that their binding areas overlap, confirming the interpretation of the multifrequency STD NMR results with kanamycin described above.

## Conclusion

Here, we have demonstrated that multifrequency STD NMR experiments are extremely sensitive spectroscopic tools to: 1) confirm binding of small molecules to bacterial biofilm-forming exopolysaccharides, as that produced by *B. multivorans* (EpolC1576), and 2) provide structural details at an atomic level of the interactions as well as average information about the orientation and area of interaction of the small molecule within the different regions of the exopolysaccharide.

It is well known that bacterial recalcitrance is mediated through several mechanisms. In this study, we have investigated the contribution and extent of involvement in recalcitrance of the exopolysaccharide EpolC1576 produced by *B. multivorans*. Confronting the data between the two antibiotics, two general but very different mechanisms can be proposed as models to explain EpolC1576 contribution to recalcitrance. The first one, regarding kanamycin, suggests that EpolC1576 might contribute to recalcitrance toward this antibiotic by direct interaction with it. The second one, regarding ceftazidime, suggests another type of mechanism involving an increase in the ceftazidime degradation rate where the role of EpolC1576 seems to be fundamental.

This proof of principle of the applicability of multifrequency STD NMR to interactions of small molecules with biofilm exopolysaccharides is of high relevance to studies to come about the structural details behind the impact of interactions of biofilm forming exopolysaccharides with small molecules, such as nutrients or drugs, on bacterial recalcitrance, where STD NMR approaches open a door to access atomic detail information on the binding modes and areas of interactions of the ligands.

## Data Availability

The raw data supporting the conclusion of this article will be made available by the authors, without undue reservation.
