# Supplementary material for: Multifrequency STD NMR Unveils the Interactions of Antibiotics With Burkholderia multivorans Biofilm Exopolysaccharide
Source: Front Mol Biosci. 2021 Sep 16;8:727980. doi: 10.3389/fmolb.2021.727980 (PMC8481691; doi:10.3389/fmolb.2021.727980)
Supplement: Supplementary file 1 [file DataSheet1.pdf]

SUPPORTING INFORMATION

Multifrequency-STD NMR unveils the Interactions of Antibiotics with *Burkholderia multivorans* Biofilm Exopolysaccharide

Ridvan Nepravishta[1], Serena Monaco[1], Marco Distefano[2], Roberto Rizzo[2], Paola Cescutti[2] and Jesus Angulo[1,3,4,*]

[1] School of Pharmacy, University of East Anglia, Norwich Research Park, NR4 7TJ, Norwich, UK

[2] Department Life Sciences, University of Trieste, Via Licio Giorgieri 1 34127 Trieste, Italy.

[3] Department of Organic Chemistry, Faculty of Chemistry, University of Seville, 41012 Seville, Spain

[4] Instituto de Investigaciones Químicas (CSIC-US), Avda. Américo Vespucio, 49, 41092 Seville, Spain

*** Correspondence:**
Jesus Angulo
j.angulo@uea.ac.uk; jangulo@us.es

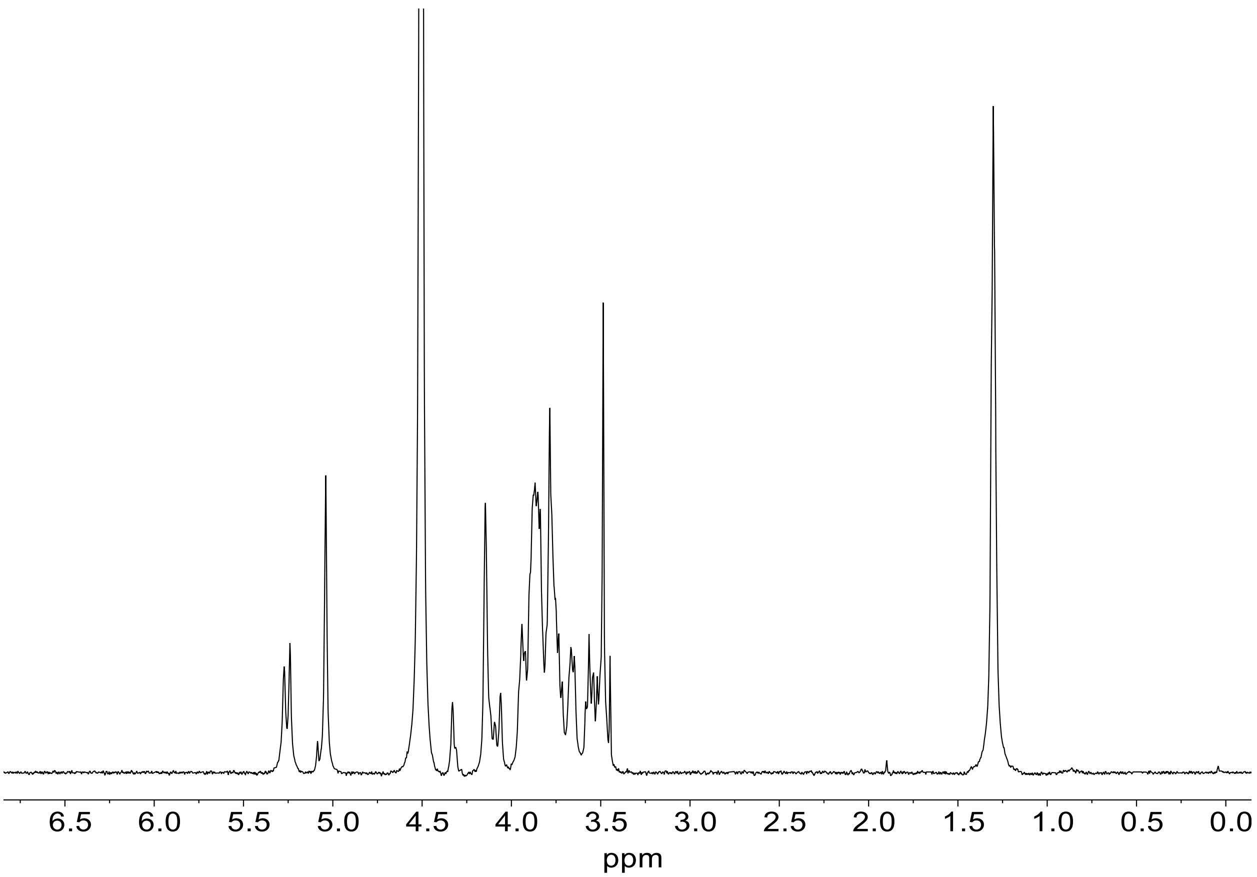

Figure S1.- Purity of the EpolC1576 sample used for the STD NMR study. ^{1}H NMR spectrum (50 °C, 500 MHz) of EpolC1576 in D_2O. Only signals from the exopolysaccharide are observable, and the integral values of the anomeric proton peaks and rhamnose methyl groups are in perfect agreement with the structure, confirming the purity of the sample.

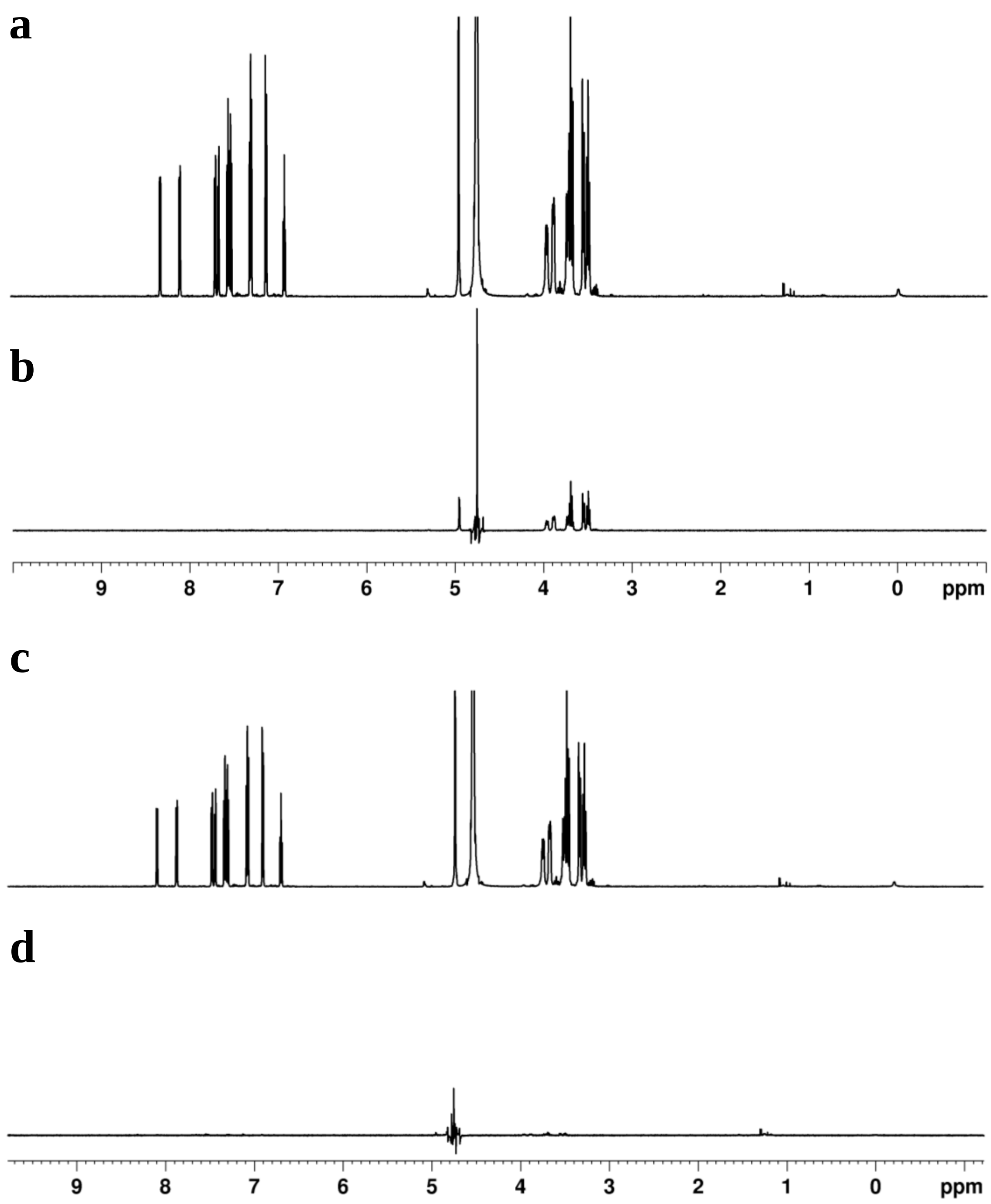

Figure S2.- STD NMR of control sample ANS + dextran. Reference 1H NMR spectra (a and c) and STD NMR spectra with irradiation at 3.22 ppm (b) or 1.22 ppm (c) of the sample of ANS in the presence of dextran (600 MHz, 5 mM ANS, 0.5 mg/mL of dextran, 298 K). The absence of STD NMR signals in the aromatic regions confirm that ANS is not binding to dextran, in contrast to the exopolysaccharide EpolC1576.
